# Adenoid cystic carcinoma of the sublingual gland developing lung metastasis 20 years after primary treatment: A case report and literature review

**DOI:** 10.1097/MD.0000000000028098

**Published:** 2021-12-10

**Authors:** Keiichi Ohta, Shinpei Matsuda, Akitoshi Okada, Masato Sasaki, Yoshiaki Imamura, Hitoshi Yoshimura

**Affiliations:** a Department of Dentistry and Oral Surgery, Unit of Sensory and Locomotor Medicine, Division of Medicine, Faculty of Medical Sciences, University of Fukui, Fukui, Japan; b Department of Thoracic Surgery, Unit of Surgery, Division of Medicine, Faculty of Medical Sciences, University of Fukui, Fukui, Japan; c Division of Surgical Pathology, University of Fukui Hospital, Fukui, Japan.

**Keywords:** adenoid cystic carcinoma, distant metastasis, follow-up, lung metastasis, salivary gland tumor, sublingual gland

## Abstract

**Rationale:**

Adenoid cystic carcinoma (ACC) is a rare malignant tumor that primarily occurs in the salivary glands. Distant metastases can develop despite favorable local control. Moreover, distant metastasis of ACC can occur after a long time interval without local recurrence. We report the first case of ACC of the sublingual gland that developed lung metastasis 20 years after primary treatment.

**Patient concerns:**

A 52-year-old man was referred to our department with a 1-year history of painful swelling on the right oral floor.

**Diagnosis:**

An incisional biopsy was performed, and histopathological examination revealed malignancy.

**Interventions:**

Surgical excision of the right oral floor and right supra-omohyoid neck dissection with postoperative chemoradiation therapy were performed, and ACC of the sublingual gland was diagnosed. Left pulmonary metastasis was detected 20 years after the primary treatment. Metastasectomy was performed; however, subsequently, skin and bone metastases developed.

**Outcomes:**

After receiving palliative care, the patient died of multiple organ failure.

**Lessons:**

As late distant metastasis of salivary ACC can develop, patients who undergo primary treatment need a long-term, strict follow-up plan even if locoregional control is favorable.

## 1 Introduction

Adenoid cystic carcinoma (ACC) is a rare malignant tumor that primarily occurs in the salivary glands.^[1]^ It accounts for 1% of the cancers of the head and neck^[1]^ and 5% to 10% of malignant salivary gland tumors.^[1,2]^ ACC mostly develops in middle-aged individuals (40–60 years) with a female predominance.^[3]^ In the salivary gland ACC, the minor salivary glands (40%–60%) are the most affected.^[4,5]^ As for the major salivary glands, the most predominant site of ACC is the parotid gland (21%–32%), followed by the submandibular gland (13%–23%), and the sublingual gland (1%–4%).^[4,5]^ ACC is characterized by slow, indolent growth, perineural invasion, and late hematogenous distant metastasis.^[1,6]^ The common clinical symptoms are a slowly growing mass, mild pain, and paresthesia.^[1,7]^ The duration of these symptoms ranges from months to several years.^[7]^ The most predominant site of distant metastasis is the lung, followed by the bone and liver. ^[1]^ The survival rates at 5-, 10-, 20-, and 25-years are 68%, 52–65%, 27–28%, and 20%, respectively.^[8–10]^ Distant metastasis of ACC results in a significantly lower survival rate.^[1]^ The 5-year locoregional recurrence rate is approximately 40%, and the rate of distant metastasis ranges from 8 to 60%.^[11]^ The average time of metastasis is 5 years although the cases with over a decade exist.^[12]^ Distant metastasis can develop despite a favorable local control.^[13–15]^ Moreover, distant metastasis of ACC can occur after a long time interval without local recurrence.^[16–21]^ Jaso and Malhotra suggested that primary ACC and metastatic ACC should be treated as different lesions.^[14]^ In this regard, managing ACC with distant metastasis is challenging for the clinician. Herein, we report the first case of ACC of the sublingual gland developing lung metastasis 20 years after primary treatment. We also review the literature on cases of ACC developing distant metastasis after a long-term interval.

## 2 Case report

**Figure 1:**
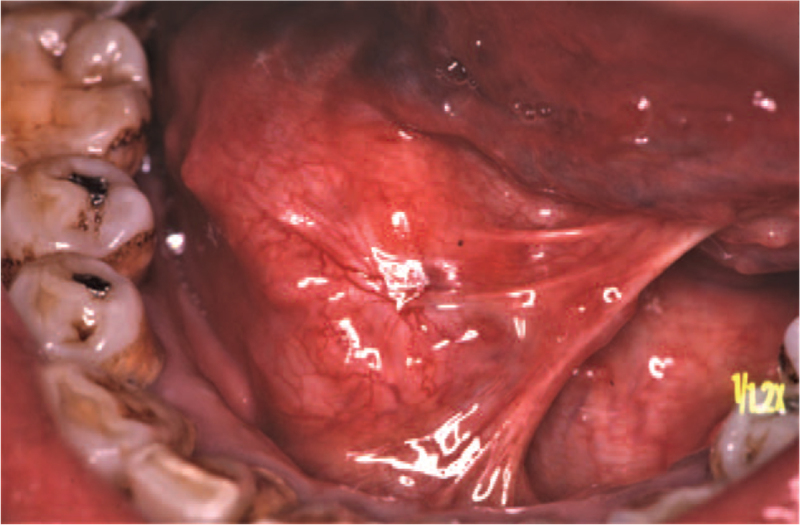
Intraoral examination showing a solitary, elastic hard, spherical 45 × 30 mm submucosal mass and paresthesia on the right oral floor.

**Figure 2:**
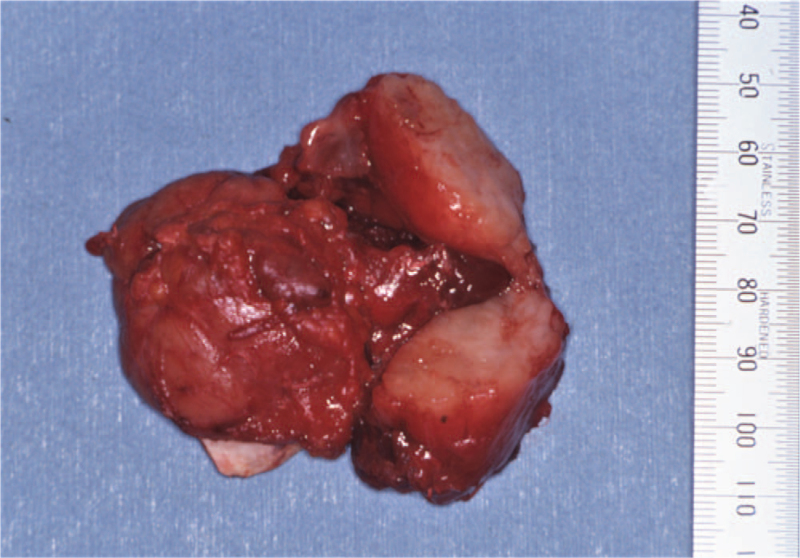
The resected specimen showing a solid mass with surrounding soft tissues. The mass was filled with white, uniform material.

**Figure 3:**
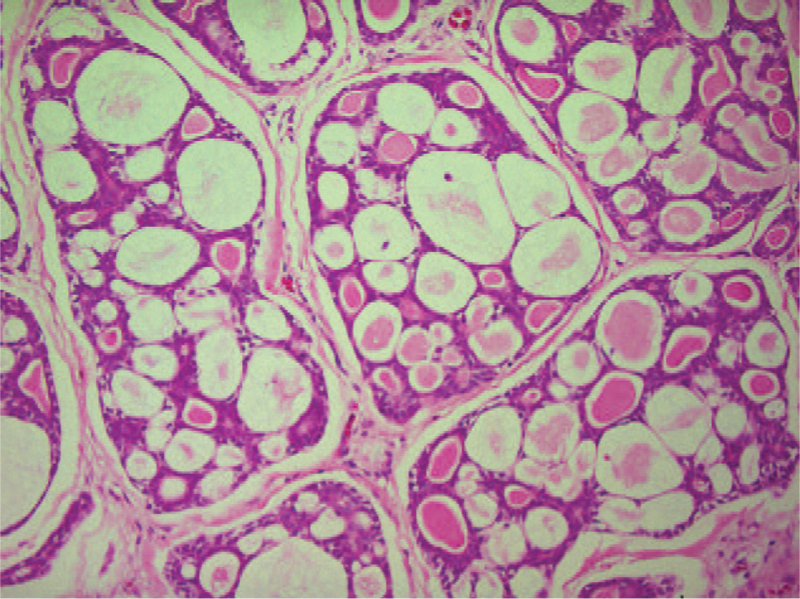
Histopathological findings showing islands of basaloid cells surrounding cyst-like spaces (pseudocysts), revealing a cribriform pattern, hematoxylin and eosin stain; magnification, × 100.

**Figure 4:**
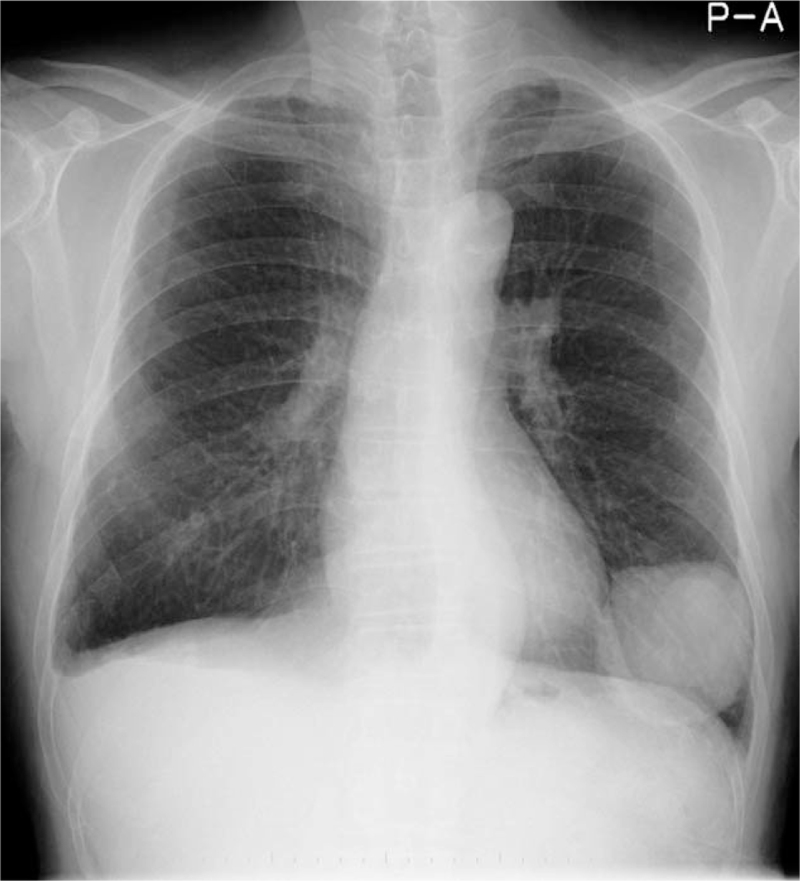
Chest radiograph showing a rounded mass shadow in the left lower lobe.

**Figure 5:**
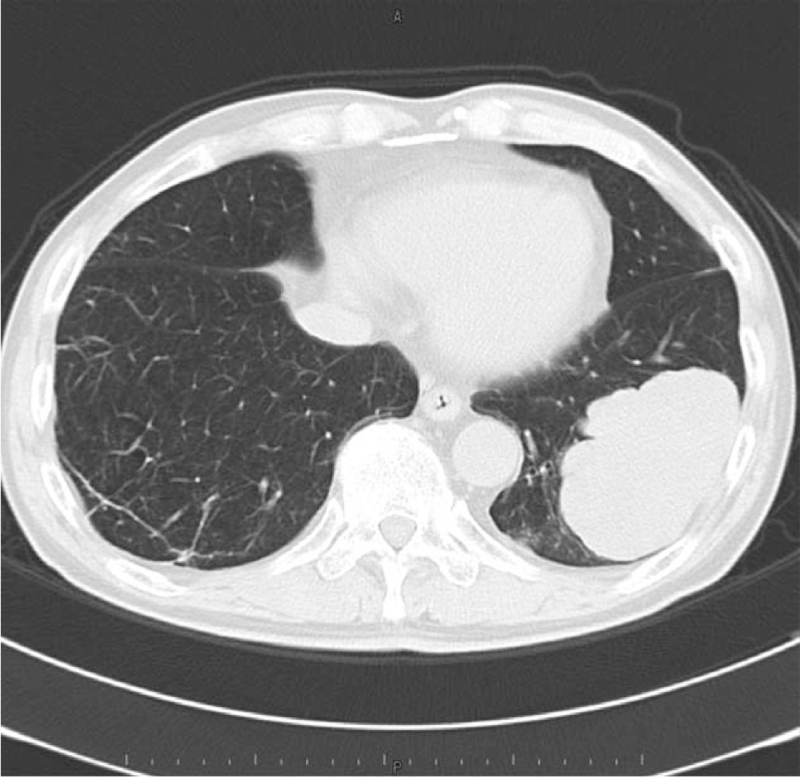
Axial chest computed tomography (CT) (lung window setting) showing 8.5 cm mass shadow in the left lower lobe, and many flat nodules along with the left pleura.

**Figure 6:**
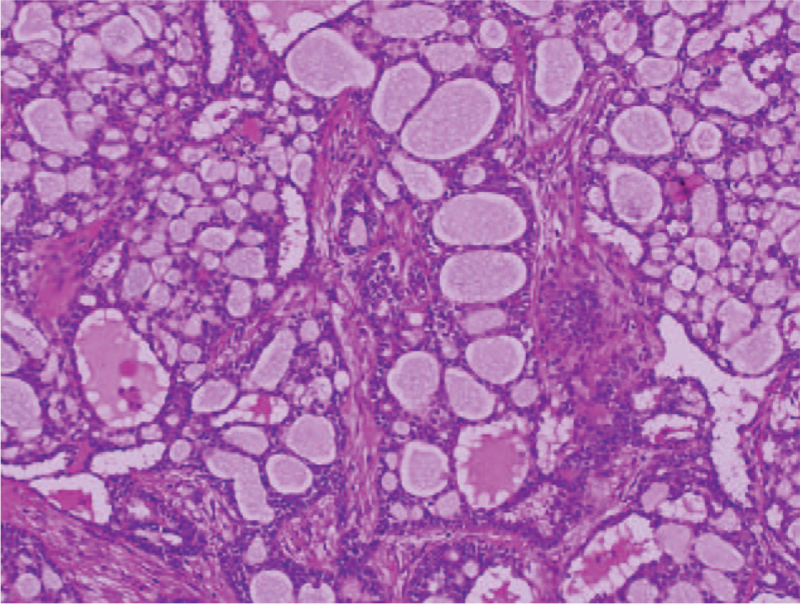
Histopathological findings of resected lung tumor showing a histological pattern similar to the sublingual gland, excised 20 years back, hematoxylin and eosin stain; magnification, × 100.

A 52-year-old man was referred to our department in January 1991, with a 1-year history of painful swelling in the right oral floor. He reported that he had undergone excision of the mass at the same location, which had been clinically diagnosed as a ranula by a dentist 8 years earlier (in 1983). After treatment, he was aware of a painless swelling on the right oral floor; however, it was left untreated. His medical history included gastric ulcers, and his mother had a history of tongue cancer. Intraoral examination of the patient revealed a solitary, elastic hard, spherical 45 × 30 mm submucosal mass, and paresthesia on the right oral floor (Fig. 1). Extraoral examination revealed no cervical lymphadenopathy. Imaging examination showed no evidence of cervical or distant metastases. An incisional biopsy was performed, and histopathological examination revealed malignancy. Therefore, he underwent surgical excision of the right oral floor and right supra-omohyoid neck dissection under general anesthesia in February 1991 (Fig. 2). Histopathological examination of the specimen confirmed ACC in the sublingual gland (Fig. 3). No metastases were observed in the dissected lymph nodes. He received postoperative radiotherapy (50 Gy) and chemotherapy with two cycles of carboplatin (450 mg/day) for 1 day and 5- fluorouracil (1,000 mg/day) for 5 days. There was no evidence of recurrence on computed tomography (CT) at 3 months follow-up. He was followed up regularly with periodical chest radiographs until August 1997; however, he was lost to follow-up. In August 2011, he was referred to our institution with left-sided chest pain and coughing. The chest radiograph revealed a rounded mass shadow in the left lower lobe (Fig. 4). A whole CT scan revealed an 8.5 cm mass shadow in the left lower lobe, and many flat nodules along with the left pleura (Fig. 5). ^18^F-fluorodeoxyglucose positron emission tomography/CT scans showed increased uptake within the mass in the left lower lobe. No evidence of local recurrence of the oral region was found. Based on these findings, a clinical diagnosis of left lower lobe lung cancer was made. Video-assisted thoracoscopic lobectomy of the left lower lobe with lymph node dissection and intraoperative hyperthermic intrathoracic perfusion chemotherapy was performed under general anesthesia in August 2011. Histopathological examination confirmed ACC, which was compatible with the specimen of the sublingual gland excised 20 years ago (Fig. 6). The patient was diagnosed with metastatic ACC. During follow-up, the patient presented with a left chest subcutaneous nodule that increased in size. Surgical excision of the left chest subcutaneous nodule was performed under local anesthesia in January 2013. Histopathological examination revealed ACC. Subsequently, adjuvant chemotherapy was initiated. In April 2013, CT and magnetic resonance imaging revealed metastasis to the thoracic vertebrae. Radiotherapy was also initiated in the same month; however, chemoradiation therapy was discontinued because his general condition deteriorated in the following month. After being transferred to the palliative care unit, the patient died due to multiorgan failure in July 2013. Informed consent was obtained from the patient's relatives for publication of the case and the accompanying images.

## 3 Discussion

To the best of our knowledge, only nine cases of oral and salivary gland ACC developing distant metastasis more than 10 years after initial treatment have been reported in the literature (Table 1).^[17–25]^ The mean age at primary treatment was 39 years (15–61 years), and the male-to-female ratio was 3:2. The most common location of the primary ACC was the submandibular gland (40%), followed by the parotid gland (20%), tongue, lower lip, gingiva, and sublingual gland (10%). Surgical resection was performed for primary ACCs, except for one case (90%); among them, in 67% of cases surgical resection was combined with adjuvant radiation or chemoradiation therapy. The most common histopathological type was cribriform (30%) and a mixed pattern of tubular and cribriform (30%), followed by tubular (20%). No solid patterns have been reported. Distant metastasis without local recurrence occurred in 60% of cases. The most predominant site of distant metastasis was the lung (80%), and 30% of patients developed distant metastasis more than 20 years after the initial treatment. Surgical treatment for metastatic tumors was performed in 50% of cases, whereas radiation or chemotherapy without surgery was performed in 20% of cases. No treatment was performed in 20% of cases.

**Table 1:** Oral and salivary gland adenoid cystic carcinoma developing distant metastasis more than 10 years after initial treatment.

Case	1	2	3	4	5	6	7	8	9	10
Author (year)	Ohara et al.^[17]^ (2007)	Yeung et al.^[22]^ (2009)	Falk et al.^[18]^ (2011)	Coupland et al.^[23]^ (2014)	Liu et al.^[24]^ (2015)	Rafael et al.^[25]^ (2016)	Portilla et al.^[19]^ (2018)	Kamigaichi et al.^[20]^ (2018)	Morassi et al.^[21]^ (2020)	Present case (2021)
Age/ sex	27/ F	23/ F	49/ M	15/ F	41/ M	52/ M	61/ M	45/ F	25/ M	52/ M
Primary location/ metastasis	SMG/ NA	SMG/ NA	Tongue/ NA	SMG/ NA	PG/ NA	SMG/ NA	Lower lip/ NA	Gingiva/ NA	PG/ intraparotid LN	SLG/-
Size (mm)	NA	NA	NA	NA	NA	25	NA	NA	25	45
Initial treatment	Resection	Resection	Resection, RT	Resection, CRT	Resection, ND, RT	Resection, RT	Resection, RT	NA	Resection, ND	Resection, ND, CRT
Histological subtype	NA	Cribriform^∗^	NA	Tubular, cribriform^∗^	Tubular	Cribriform	Tubular	Tubular, cribriform	Tubular, cribriform	Cribriform
Recurrence/ time to recurrence	–	+/ 5, 10 yr	-	+/ 35 yr	+/ 9 yr	+/ 9 yr	-	-	-	-
Time to metastasis/ location	14 yr/lung	10 yr/lung	10 yr/ lung, 15 yr/ pancreas	38 yr/liver	10 years/ lung	10 yr/ lung, tongue, 12 yr /toe	11 yr/ lung, liver, skin, brain, choroid	5 yr/ cervical LN, 36 yr/ lung	25 yr/brain	20 yr/ lung, 22 yr/ skin, vertebrae
Treatment for metastasis	None ^†^	None ^†^	Surgery	Surgery	NA	CRT	RT	Surgery	Surgery, RT	Surgery, CRT
Prognosis after treatment for metastasis/ yr	Died with disease/4 yr	Alive with disease/4 yr	No evidence of disease/ 0.5 yr	No evidence of disease/ 0.5 yr	NA	Alive with disease/1 yr	NA	No evidence of disease/2 yr	NA	Died with disease/ 2 yr

Our review showed that no solid pattern of ACC with late metastasis has been reported. ACC has three histopathological patterns: cribriform, tubular, and solid,^[1]^ and of these, the solid pattern is recognized as a high-grade tumor and associated with poor prognosis.^[10,26]^ Ishida et al reported that early distant metastasis could result from a solid pattern.^[10]^ ACC with cribriform and/or tubular pattern has a better prognosis than the solid pattern^[10,26]^; however, long-term careful observation may be desirable after initial treatment of ACC with cribriform and/or tubular patterns to detect delayed recurrence and distant metastasis.

In our review, in 60% of cases of ACC, the patients developed late distant metastasis without local recurrence, and in 80% of cases the patients had metastasis to the lung. The risk factors for lung metastasis include tumor size, perineural invasion, and local recurrence.^[27]^

Since primary lung ACC is extremely rare, the possibility that the metastatic tumor might be metachronous multiple tumors is highly unlikely.^[28]^ In a 10-year retrospective observational study by Oplatek et al, 42% of patients with distant metastasis had no evidence of locoregional failure.^[29]^ In addition, metastatic ACC of the head and neck, particularly lung metastasis, is reported to remain asymptomatic for a long time.^[15,30]^ According to the literature, the mean tumor doubling time of metastatic ACC to the lung is long (86–1064 days with an average of 393 days).^[31]^ Kamiyoshihara et al reported that the tumor doubling time of resected metastatic lung tumors with a disease-free interval greater than 10 years was 80–815 days.^[32]^ They suggested that some lung tumors might require several decades to become detectable radiologically.^[33]^

In the sublingual gland, ACC and mucoepidermoid carcinoma are the predominant malignant tumors.^[34]^ Seok et al reported that sublingual and minor salivary gland ACCs have a higher risk of 10-year lung metastasis rate (69.8%) than parotid and submandibular gland ACCs (28.4%).^[27]^ They also reported that sublingual and minor salivary gland ACCs tend to have a larger tumor size, higher ratio of perineural invasion, and local recurrence. Although the underlying mechanisms remain unclear, some clinical and anatomical characteristics may be responsible for the high aggressiveness of sublingual gland tumors. Sublingual gland tumors tend to be diagnosed at an advanced stage because of their relatively asymptomatic clinical course.^[16]^ Additionally, compared to parotid and submandibular glands, the sublingual gland has some anatomical features such as poor encapsulation and numerous ducts opening into the oral floor, which can lead to easier infiltration to surrounding soft tissues.^[16]^ These clinical features may lead to a higher risk of distant metastasis compared to tumors in other major salivary glands.

Radical surgical resection with postoperative radiotherapy is recognized as the standard treatment for primary ACC.^[1]^ Regarding definitive radiotherapy for major salivary glands, a recent large German multicenter study showed that radiotherapy in bimodal conditions including intensity-modulated radiotherapy and dose-escalation with carbon ion, increases the favorable local control.^[35]^ The role of prophylactic neck dissection for the N0 cases in ACC is still debated.^[36]^ Ning et al performed a systematic review and reported that the average rate of occult metastasis in salivary gland ACC was 14%.^[37]^ Xiao et al reviewed the United States National Cancer Database and reported that cN0 patients with ACCs in the major salivary glands and tongues had significantly increased risk of occult metastasis, and they suggested that prophylactic neck dissection is necessary for this group.^[38]^ In contrast, a recent multi-institutional retrospective analysis in Japan showed that prophylactic neck dissection for the salivary glands ACC was not associated with better clinical outcomes in the 5 year observation periods.^[39]^ Garg et al reviewed the literature and reported that prophylactic neck dissection for N0 cases in ACC can provide accurate cancer staging, prognostic prediction, and locoregional control, even if it does not contribute to overall survival.^[36]^ With regard to the significance of postoperative radiotherapy, no consensus has been reached yet.^[4,40,41]^ Garg et al also reported that postoperative radiotherapy improves locoregional control; however, in patients with clear margins, small tumor, and no adverse features, postoperative radiotherapy can lead to adverse effects.^[36]^ The effect of metastasectomy has remained controversial; however, recent retrospective studies with long-term observation showed that lung metastasectomy resulted in longer survival. ^[10,42,43]^ Girelli et al suggested that lung metastasectomy should be considered if the disease-free interval is greater than 36 months and complete resection of metastatic lesions is feasible.^[43]^ Their study also showed that there was no significant difference in survival rate in patients with single or multiple metastatic lesions in the lung.

Due to the slow-glowing characteristic of recurrent/metastatic ACC, chemotherapy is poorly effective.^[42]^ In the field of molecular biology, Ho et al reported that in recurrent/metastatic ACC, Notch signaling, chromatin-remodeling pathways, and the telomerase reverse transcriptase promoter are highly altered.^[44]^ The advancement of genetic profiling of ACC may promote the development of effective targeted therapies against metastatic ACC.

As for immunotherapy, molecular and histological features of ACC suggest that it has low immunogenicity.^[42]^ However, the role of immunotherapy is under investigation and needs further studies.

The reported clinicopathological prognostic factors of salivary gland ACC are higher T and N classification, higher solid pattern components, and pathologically positive surgical margins.^[4,39,45]^

Since ACC grows in an indolent clinical course beyond 20 years with a high recurrence and metastatic rate,^[10]^ a detailed follow-up strategy is necessary. Several studies suggest that the practical major goal of treatment of ACC maybe long-term survival with a cancer-baring state, rather than concluding the disease as cured.^[1,10]^ Ishida et al suggested that unlimited follow-up observations may be desirable.^[10]^ Garg et al suggested that periodical chest CT may be more suitable for detecting early lung metastasis as chest radiographs are not sufficiently sensitive.^[36]^^18^F-fluorodeoxyglucose positron emission tomography/CT scan is unable to rule out distant metastasis as ACC absorbs less FDG, and it might be difficult to discern from the normal physiological uptake in salivary glands.^[1]^ Garg et al reported that chest CT or cross-sectional imaging of the full-body may have a role in long-term observation while considering the risk of developing radiation-induced tumors.^[36]^ Although 8 years have passed since the patient's death, the patient was treated in much the same way as the treatment indicated in the current guidelines.^[46]^ Nevertheless, distant metastasis occurred 20 years after primary treatment without local recurrence. To date, no guidelines about follow-up strategy for salivary gland cancer, especially ACC are available.^[46]^ We believe that our case provides useful findings about the importance of extremely long-term follow-up in salivary gland ACC.

In conclusion, we reported a case of a patient with ACC of the sublingual gland that developed lung metastasis 20 years after primary treatment. Since late distant metastasis of salivary ACC can develop, patients who undergo primary treatment need a long-term, strict follow-up plan even if locoregional control is favorable.

## Author contributions

**Conceptualization:** Keiichi Ohta, Hitoshi Yoshimura.

**Data curation:** Shinpei Matsuda, Yoshiaki Imamura.

**Investigation:** Shinpei Matsuda, Akitoshi Okada.

**Resources:** Masato Sasaki, Yoshiaki Imamura.

**Supervision:** Masato Sasaki, Hitoshi Yoshimura.

**Visualization:** Akitoshi Okada, Yoshiaki Imamura.

**Writing – original draft:** Keiichi Ohta.

**Writing – review & editing:** Hitoshi Yoshimura.
